# How much radiologist time can be saved by implementing AI in screen-reading mammograms?

**DOI:** 10.1007/s00330-025-12273-x

**Published:** 2026-01-07

**Authors:** Tone Hovda, Åsne S. Holen, Solveig Hofvind

**Affiliations:** 1 Department of Radiology, Vestre Viken Hospital Trust, Drammen, Norway; 2 Department of Breast Screening, Cancer Registry, Norwegian Institute of Public Health, Oslo, Norway; 3 Department of Health and Care Sciences, Faculty of Health Sciences, UiT The Arctic University of Norway, Tromsø, Norway

**Keywords:** Early detection of cancer, Breast cancer, Mammography, Artificial intelligence (AI)

## Abstract

**Objective:**

There is a lack of breast radiologists in Norway and in Europe. Artificial intelligence (AI) offers an alternative to solely human readers and has demonstrated promising results in cancer detection in mammographic screening. We aimed to estimate the potential reduction in radiologists’ workload by replacing one of the two radiologists with AI in screen-reading mammograms in BreastScreen Norway.

**Materials and methods:**

BreastScreen Norway targets about 680,000 women aged 50–69 who are invited biennially. The participation rates for each screening round are about 75%. All the screening mammograms are independently read by two radiologists.

We collected information about the number of radiologist positions from all 16 breast centers in the country in 2024, while the number of screening examinations performed and the time spent on screen-reading and consensus were extracted from the screening database. We used 1 min for each screen-reading to estimate the screen-reading workload performed by the radiologists and calculated the time saved if one reader were replaced by AI.

**Results:**

Screen-reading required a total of 6.5 man-years in BreastScreen Norway. Implementing AI as one of the two readers is thus able to reduce the screen-reading workload by 50%, from 6.5 to 3.3 man-years. The workload reduction corresponds to a reduction from 9% to 4.5% of the total workload for radiologists at the Norwegian breast centers.

**Conclusion:**

Implementation of AI in mammographic screening has the potential to reduce the screen-reading workload for breast radiologists. The reduced screening volume is of moderate influence on the overall workload for breast radiologists.

**Key Points:**

***Question*** *How much radiology time is expected to be saved if AI were used as one of the two readers of screening mammograms?*

***Findings**** Use of AI as one of the two readers, reducing the screen reading volume by 50%, was of moderate influence on the total workload for breast radiologists*.

***Clinical relevance**** Implementing AI was shown to have limited potential in saving radiologists’ time in screen-reading mammograms. The main benefit of implementing AI in screen-reading might thus be related to increased sensitivity of the screening test*.

**Graphical Abstract:**

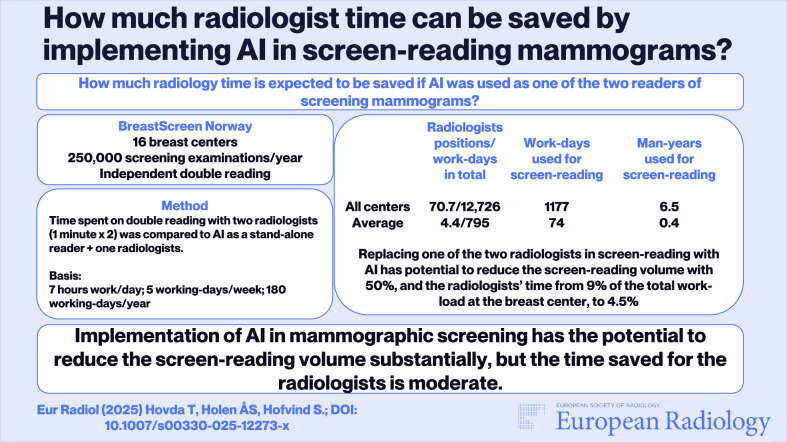

## Introduction

Mammographic screening is a cornerstone of early breast cancer detection, aimed at reducing disease-specific mortality and the burden of treatment through timely intervention [1, 2]. The standard protocol in most organized screening programs in Europe, including BreastScreen Norway, involves double reading of mammograms by two radiologists [1, 3]. In instances where both readers assign a positive score or when there is discordance between their assessments, a consensus or arbitration process is usually initiated to determine the outcome of the screening examination. This approach enhances diagnostic accuracy and helps maintain low and acceptable recall rates; however, it is resource-intensive and contributes to the growing strain on radiology services, particularly in the context of a global shortage of qualified radiologists [4].

Artificial intelligence (AI) has emerged as a promising adjunct or alternative to human readers in mammographic screening [5–8]. Several studies, retrospective as well as prospective, have demonstrated that AI systems can achieve diagnostic performance comparable to that of radiologists, particularly in terms of cancer detection and recall rates. One application is the replacement of one of the two radiologists with an AI system, either fully or partially, for example, by stratifying cases into low- and high-risk categories prior to human reading. Such integration has the potential to substantially reduce the reading workload without compromising diagnostic performance in mammographic screening.

Despite the growing body of evidence supporting the efficacy of AI in mammographic screening, relatively little attention has been paid to its cost-effectiveness and the extent of the potential for reducing radiologist workload. AI models are particularly well-suited for triaging mammograms based on estimated cancer risk, but the effectiveness of such triage depends on the selection of appropriate risk thresholds. These thresholds should be evidence-based and may need to be tailored according to incidence, individual-level factors (e.g., age, ethnicity, breast density), technical variables (e.g., mammography equipment, AI model), and contextual factors (e.g., radiologist expertise and local screening protocols) [9, 10].

AI may be deployed in various roles within the screening workflow, including as a stand-alone reader, as a decision-support tool for radiologists, or as an aid in the consensus or arbitration processes. While AI support in consensus discussions may help maintain specificity, it is unlikely to replace the need for human arbitration or significantly reduce the associated workload.

Given the increasing demand for radiological services and the limited availability of trained personnel, reducing radiologists’ workload is a compelling rationale for the adoption of AI in mammographic screening. The objective of this study was to estimate the potential reduction in radiologist workload achievable and thereby radiologists’ time saved by substituting one of the two readers with an AI model in the context of BreastScreen Norway.

## Material and methods

No individual data were used, only aggregated data on the number of examinations, recalls, and cancers, in addition to radiologist positions at the different centers in BreastScreen Norway. The data were disclosed on a legal basis in the Cancer Registry Regulations section 3–1 and the Personal Health Data Filing System Act section 19a to 19h [11, 12].

BreastScreen Norway is a population-based screening program targeting about 680,000 women aged 50–69 [13]. The program is administered by the Cancer Registry, at the Norwegian Institute of Public Health. Women are invited to undergo two-view digital mammography biennially. All screening mammograms are interpreted independently by two breast radiologists [13]. Each breast is given a score between 1 and 5, where 1 indicates negative for malignancy; 2, probably benign; 3, intermediate suspicion of malignancy; 4, probably malignant, and 5, high suspicion of malignancy. All examinations with a score of 2 or higher by one or both radiologists are discussed in a consensus meeting to determine whether the woman should be recalled for further assessment. In 2024, the attendance rate in the program was 77%, consensus 7.2%, recall 3.2%, and screen-detected cancer 0.63%.

### Study data

To explore the potential savings of implementing AI in BreastScreen Norway, we collected information about the number of radiologist positions at all 16 breast centers in Norway in 2024.

Information about the actual number of screening examinations, time used on initial interpretation, and the number of recalls was extracted from the screening database. Screen-reading involves two radiologists. Mean and median time spent on screen-reading was available for 15 of the 16 breast centers. Reading times were not available for one center due to technical issues. Mean and median values for all centers were used as the estimate for the center without a value. The duration of the 441,276 primary readings with information about reading time available ranged from 0 s to 4 h and 47 min, resulting in a mean time of 48 s and a median time of 25 s. A value of 0 was present for 289 (0.1%, 289/441,276) cases, which represent a registration error. We excluded values higher than 10 min (0.6%, 2792/441,276) as we assumed that the radiologist had been interrupted, distracted, or had forgotten to log out if the time was longer. This exclusion resulted in a mean reading time of 41 s and a median time of 25 s for the 219,242 mammography examinations and in 438,484 screen-readings. To avoid underestimation of the time spent on screen-reading, we used 1 min for initial interpretation (1 min × 2 radiologists). Mean and median time spent on consensus/arbitration were 2:12 and 1:55 min per examination [14]. Two, sometimes three radiologists are involved. To ensure no underestimation, we used 5 min multiplied by 2.5 radiologists for time spent on consensus. Time spent on recall assessment after a positive screening test was set as an average of 60 min work by one radiologist, based on the time schedule at the breast centers in Norway. The times reported for the various tasks exceed the measured values. This approach was adopted to avoid underestimating actual time, given the substantial variability and the fact that many activities related to primary reading, consensus discussions, and follow-up reviews may occur outside the formally recorded intervals.

We used five working days per week, seven working hours per day, and 180 working days per year to estimate man-years of work [15]. The estimates were based on typical working conditions in Norwegian breast radiology departments, assuming five working days per week and seven working hours per day. To estimate man-years, we used 180 working days per year instead of the standard 226 to account for absences related to professional discussions, educational activities, breaks, and sick leave. This adjustment reflects an empirically reasonable estimate derived from observed work patterns rather than a theoretical maximum. To test the robustness of our results, we also examined alternative scenarios using 200 and 226 working days per year.

### Statistics

We used data from 2024 and estimated the number of working days by multiplying the number of radiologist positions reported from each center by 180, considering the results as the total number of man-years performed at each breast center and in total. Time spent on screen-reading was calculated by multiplying 2 min per screening examination by the total number of examinations. The total number of man-years was obtained by multiplying the number of radiologist positions by 180, while the man-years spent on screen-reading were estimated by converting the total hours spent on screen-reading during a 7-h working day into man-years, and then dividing by the total number of radiologist positions to obtain the percentage of man-years dedicated to screen-reading. Man-years spent on consensus and recall were estimated in a similar manner. To explore the sensitivity of our estimates, additional calculations were provided using a reading time of 30 s per examination, 200, and 226 working days per year.

We did descriptive statistics, presenting data as medians and average times spent on screen-reading. To answer the aim of the study, we included information on time spent on screen-reading as we considered this effort to have the potential to be directly affected by AI. Additionally, the potential impact of AI in screen-reading on consensus/arbitration and recall rates, both in terms of reduction and increase, was estimated across various scenarios, based on the time assumptions described.

## Results

All 16 breast centers in Norway handed in information about the number of radiologist positions at their center. The total number of radiologists involved in screen-reading in BreastScreen Norway in 2024 was 95. These radiologists covered 70.7 positions in total (Table 1). The centers differed substantially in the number of radiologist positions (range 1.0–10.3) and the number of women screened (range 5635–24,857).

**Table 1:** Number of radiologist positions, workdays available, screening examinations performed, workdays spent on screen-reading, percent of the total work spent on screen-reading, and man-years used for screen-reading for each of the 16 breast centers and in total for BreastScreen Norway, 2024

Breast center	Radiologist positions (*n*)^*^	Workdays available in total	Screening exams performed in 2024 (*n*)	Workdays related to screening	Workload related to screening (%)	Man-years used for screening
A	7.0	1260	24,580	117	9%	0.7
B	3.0	540	12,729	61	11%	0.3
C	2.6	468	13,099	62	13%	0.3
D	1.0	180	5635	27	15%	0.1
E	2.0	360	12,549	60	17%	0.3
F	4.5	810	18,073	86	11%	0.5
G	3.0	540	8557	41	8%	0.2
H	7.5	1350	21,604	103	8%	0.6
I	10.0	1800	20,598	98	5%	0.5
J	3.0	540	15,111	72	13%	0.4
K	2.0	360	15,935	76	21%	0.4
L	7.0	1260	20,632	98	8%	0.5
M	2.0	360	10,781	51	14%	0.3
N	2.8	504	8773	42	8%	0.3
O	3.0	540	13,609	65	12%	0.4
P	10.3	1854	24,857	118	6%	0.7
Total	70.7	12,726	247,122	1177	9%	6.5
Average	4.4	795	15,445	74		0.4

^*^ Number of available radiologist positions—not all positions were necessarily filled

The radiologists at the 16 breast centers spent a total of 6.5 and an average of 0.4 man-year performing screen-reading in 2024 (Table 1). Implementing AI as one of the two readers could reduce the workload related to screen-reading by 50%, from 6.5 to 3.3 man-years, which corresponds to a reduction from 9% (1177/12,726) to 4.5% ((1177/12,726)/2) of the average total workload for the radiologists at a breast center (Table 1). For the largest breast centers, the screen-reading workload would be reduced by 0.35 man-years, from 0.7 to 0.35 by replacing one reader with AI. Additional analyses using 30 s reading time, 200 and 226 working days resulted in 3.0 and 2.6 man-years needed to screen-read 250,000 examinations.

In addition to the total of 6.5 man-years spent on screen-reading, Norwegian radiologists spent 2.9 man-years on consensus and 6.2 on recall assessments, for a total of 15.7 man-years in 2024 (Table 2). This means that screen-reading accounted for 42% (6.5/15.7) of screening-related work. Using these numbers as a value for the work spent on screening-related tasks, screen-reading and consensus counted for 13% (9.4/70.7), and if recall assessment was included, 22% (15.8/70.7) of the total man-years at the 16 breast centers in Norway.

**Table 2:** Time spent on effect measures (screen-reading, consensus, and recall) and the assumed effect of using AI, resulting in varying consensus and recall rates and man-years required by breast radiologists in Norway

Stable consensus	Values in 2024			Decreased recall			Decreased recall 2			Increased recall			Increased recall 2			Increased recall 3		
	Effect measure	Work days	Man-years	Effect measure	Work days	Man-years	Effect measure	Work days	Man-years	Effect measure	Work days	Man-years	Effect measure	Work days	Man-years	Effect measure	Work days	Man-years
Screen-reading	100%	1177	6.5	100%	1177	6.5	100%	1177	6.5	100%	1177	6.5	100%	1177	6.5	100%	1177	6.5
Consensus	7.2%	530	2.9	7.2%	530	2.9	7.2%	530	2.9	7.2%	530	2.9	7.2%	530	2.9	7.2%	530	2.9
Recall	3.2%	1130	6.3	2.8%	988	5.5	2.0%	706	3.9	3.6%	1271	7.1	4.5%	1589	8.8	5.5%	1942	10.8
Total		2837	15.8		2695	15.0		2413	13.4		2978	16.5		3296	18.3		3649	20.3
% of total man-years at the breast center			22%			21%			19%			23%			26%			29%
**Increased consensus**	**Values in 2024**			**Decreased recall**			**Stable recall**			**Increased recall**								
	**Effect measure**	**Work days**	**Man-years**	**Effect measure**	**Work days**	**Man-years**	**Effect measure**	**Work days**	**Man-years**	**Effect measure**	**Work days**	**Man-years**						
Screen-reading	100%	1177	6.5	100%	1177	6.5	100%	1177	6.5	100%	1177	6.5						
Consensus	7.2%	530	2.9	10%	735	4.1	10%	735	4.1	10%	735	4.1						
Recall	3.2%	1130	6.3	2.8%	988	5.5	3.2%	1120	6.2	3.6%	1271	7.1						
Total		2837	15.8		2900	16.1		3032	16.8		3183	17.7						
% of total man-years at the breast center			22%			23%			24%			25%						
**Decreased consensus**	**Values in 2024**			**Decreased recall**														
	**Effect measure**	**Work days**	**Man-years**	**Effect measure**	**Work days**	**Man-years**												
Screen-reading	100%	1177	6.5	100%	1177	6.5												
Consensus	7.2%	530	2.9	5.0%	368	2.0												
Recall	3.2%	1130	6.3	2.0%	706	3.9												
Total		2837	15.8		2251	12.5												
% of total man-years at the breast center			22%			18%												

Assuming a stable consensus rate of 7.2% (17,809/247,118) and a decrease in recall, from 3.2% (7840/247,118) to 2.0% (4942/247,118), the percentage of screening-related work for radiologists decreased from 22% (15.8/70.7) to 19% (13.4/70.7) (Table 2). A stable consensus rate but an increased recall rate from 3.2% (7840/247,118) to 5.5% (13,591/247,118), increased the total screening-related workload from 22% (15.8/70.7) to 29% (20.3/70.7). A consensus rate of 10% (2472/247,118) and a stable recall rate of 3.2% (7840/247,118) increased screening-related work from 22% (15.8/70.7) to 24% (16.8/70.7).

## Discussion

This study found that replacing one radiologist with AI in the double reading of screening mammograms and thereby reducing the radiologists’ screen-reading workload by 50%, has a low to moderate impact on the overall workload for the radiologists at the breast centers in Norway.

In breast centers performing mammographic screening and multimodal breast diagnostics of symptomatic women, the burden of screen-reading constitutes only a part of the radiologists’ daily tasks. Screen-reading is rapid, 30–60 s per examination, which allows for a high volume of screen-reading to be completed in a short time [14, 16]. However, time spent on screen-reading varies between the radiologists; the benefit related to the use of AI will vary accordingly.

In this study, we calculated the workload savings of replacing one of the two radiologists with AI for all examinations. Replacing one radiologist with AI is the most radical reduction in workload that AI can provide, given current European guidelines on double reading [1] and our perceptions of the AI Act’s requirement that decisions made by AI in health care should be overseen by humans [17]. However, the procedures for “overseen” used in the AI act need to be defined and might open for solely AI reading of low-risk mammograms in the future.

The potential of AI to reduce reading time remains largely unexplored. Future interpretation procedures in which two AI models replace radiologists for examinations deemed negative, or where certain examinations are triaged directly to consensus, may offer promising solutions. However, these approaches will require studies and careful evaluation to ensure diagnostic accuracy and appropriate integration into existing practice.

AI can be used for triaging into single or double reading, and further as a stand-alone reader or as decision support for the reader. The pros and cons of offering the readers access to the AI risk score and marking are discussed [9, 18–22]. Using AI as a decision support may introduce automation bias [19, 20] and be of influence on the reading time [9, 18, 22]. However, whether the reading time will be shorter, the same, or longer remains uncertain. Studies using digital breast tomosynthesis (DBT) in combination with AI have shown varying results [18, 22]. However, DBT is known to be more time-consuming compared to standard digital mammography, related to screen-reading. Given this uncertainty, we considered using the same time estimate as without AI to be an appropriate approach.

Our time estimations assume 1 min per screen-read, multiplied by two to account for the involvement of two readers. However, the median measured reading time was 25 s, and the mean 41 s, which means that some examinations required substantially longer reading time. This is as expected in a clinical setting and could be due to real differences, disruption, and small breaks. Our values may underestimate the actual time used in clinical practice. The timing measurement started when the identification of the screened woman was activated and ended when the interpretation score was recorded. To validate the use of 1 min per screen-read, we consulted several screen-readers, who confirmed the estimate as reasonable. We adjusted the measured times upwards to avoid underestimating the time required for primary reading, consensus, and further assessment. These measures should be interpreted with respect for the radiologists, considering the variability both among those performing the tasks and across the cases being read and discussed. Furthermore, an earlier version of the European guidelines for breast cancer screening recommends that 60 screening examinations should be read per hour, supporting our assumption [16].

It could be argued that it is not possible to screen-read all day, five days a week. Our estimations do not require full positions dedicated to screen-reading. The 6.5 positions could be divided into several part-time positions, which we assume would work at the actual speed.

We estimated the potential reduction in radiologist workload based on the use of AI as a second reader. If AI were used for triaging, cases assigned a low-risk score could be single-read, and high-risk cases double-read. This stratified approach introduces uncertainty in the estimation of total screen-reading time, as cases with low-risk scores may require less time to interpret, whereas high-risk cases are likely to be more complex and time-consuming. Consequently, whether the time saved by using AI in screen-reading is over- or underestimated in such a scenario depends on the threshold used to distinguish between low- and high-risk cases, and thus whether one or two readers will be assigned for screen-reading.

The time estimates presented in this study were based on digital mammography (DM). However, Digital Breast Tomosynthesis might be adopted as a screening modality and is associated with longer interpretation times compared to DM [14, 22]. The potential integration of AI as a replacement for one of the two readers may help offset some of this additional time burden. More time-intensive components of the screening process, such as consensus discussions and recall assessments, require substantial radiologist involvement. The impact of AI implementation on these specific tasks remains uncertain. Current prospective studies suggest that AI-assisted screen-reading may lead to no change or only a modest increase in recall rates [5, 6]. Our estimations indicate that an increase in recall rate from 3.2% to 5.5% would result in a rise in radiologist workload equivalent to an increase from 6.2 to 10.8 man-years. This increase surpasses the estimated reduction in workload achieved by replacing one of the two screen-readers with AI, highlighting the narrow margins for achieving net time savings through AI implementation in mammographic screening. Conversely, the use of AI as a decision-support tool may enhance radiologists’ confidence in their screen-readings.

AI-assisted mammography screening may reduce radiologist workload, but the full cost-effectiveness is sensitive to implementation and maintenance requirements [23, 24]. Licensing fees, hardware, IT integration, and the time needed for local validation, pilot testing, and ongoing quality assurance can be substantial. These tasks often involve radiologists, medical physicists, and IT staff, many of whom are already in short supply, potentially offsetting the nominal time savings. Claims of cost savings should therefore be interpreted cautiously and ideally supported by sensitivity analyses or local pilot data.

There are several limitations of this study. We did not include any cost-effective analysis, which are crucial for understanding the potential benefit of implementing AI in the screen-reading of mammograms. While workload reduction is economically beneficial, it must be balanced with the cost of implementing, maintaining, and running the AI models. However, the impact of using AI on cancer detection, and the increased sensitivity of the screening test, is shown to be promising [5–8]. These improvements indicate potential economic savings [25], as well as benefits for women and society.

The reported workload and time spent on various tasks by the breast centers were based on rough estimates rather than meticulous counting and registrations. However, the screening workload was estimated from complete data from our screening database and was comparable for all breast centers. Several breast centers reported that not all radiologists’ positions were filled. As we used the number of positions as the basis for the estimations, we might have underestimated the proportion of the workload related to screen-reading. Given that we used 1 min for each screen-reading and 5 min for each consensus, we consider the uncertainties in our estimates to be relatively balanced.

We acknowledge that the time spent on screen reading varies substantially between cases and across radiologists. However, when radiologists wish to access additional prior images or discuss a case, a score of 2 is assigned at some of the breast centers. This indicates a consensus reading and provides access to more prior images as well as time for discussion. Furthermore, individual working styles among radiologists are reflected in the variation in screen-reading times. To avoid underestimating the time required for primary reading, consensus, and further assessment, we adjusted the measured times upwards and used the median reading time in our analyses.

The data used in this study were obtained from BreastScreen Norway. However, the reading time in primary screening is expected to be broadly comparable across countries, making the time estimates for primary reading transferable. Some caution should be exercised when applying the Norwegian estimates for consensus discussions and further assessments, but with appropriate adjustments, our model may serve as a useful starting point for other settings.

To conclude, the implementation of AI in mammographic screening in programs using double reading has the potential to reduce the screen-reading workload. However, the impact on the total workload for the radiologists working at a breast center and the challenges related to workforce deficiencies remain moderate.

## Abbreviations

AI: Artificial intelligence
